# Acute stress disorder and associated factors among adult trauma patients in Ethiopia: a multi-institutional study

**DOI:** 10.1186/s12888-024-05861-6

**Published:** 2024-06-04

**Authors:** Tewodros Guay Hagos, Tadesse Tarik Tamir, Belayneh Shetie Workneh, Nega Nigussie Abrha, Negesu Gizaw Demissie, Daniel Ayelegne Gebeyehu

**Affiliations:** 1 Department of Emergency and Critical Care Nursing, School of Nursing, College of Medicine and Health Sciences, University of Gondar, Gondar, Ethiopia; 2 Department of Pediatrics and Child Health Nursing, School of Nursing, College of Medicine and Health Sciences, University of Gondar, Gondar, Ethiopia; 3 Department of Medical Nursing, School of Nursing, College of Medicine and Health Sciences, University of Gondar, Gondar, Ethiopia; 4 Department of Psychiatry, School of Medicine, College of Medicine and Health Sciences, University of Gondar, Gondar, Ethiopia

**Keywords:** Acute traumatic stress, Trauma, Adult, Ethiopia

## Abstract

**Introduction:**

Acute stress disorder (ASD) is a mental disorder that happens after someone experienced traumatic event within duration of less than a month. Other studies conducted in different countries revealed that adults with a trauma had experienced acute stress disorder. This results in substantial distress and interferes with social and day to day activities. Despite the high burden of this problem, very little is known about the prevalence and risk factors for acute stress disorder in adults with traumatic injuries in Ethiopia.

**Objective:**

This study was aimed to assess the prevalence of acute stress disorder and associated factors among adult trauma patients attending in northwest Amhara Comprehensive Specialized Hospitals, Ethiopia 2022.

**Methods:**

An institutional based cross-sectional study design was employed among 422 adult trauma patients from May– June 2022. Systematic sampling technique was applied to recruit study participants. Data were collected through interviewer administered questionnaires using the Diagnostic and Statistical Manual of Mental Disorders, Fifth Edition, acute stress disorder measurement tools. Then, it was entered into Epi-Data version 4 and exported to STATA version 14 for analysis. Bivariate and multivariable binary logistic regressions model were carried out to identify factors significantly associated acute stress disorder.

**Result:**

The prevalence of acute stress disorder among adult trauma patients in northwest Amhara comprehensive specialized hospitals was found to be 44.15% (95% CI: 39.4%, 49.0%) with 99% of response rate. In multivariate logistic analysis younger age (21–29) (AOR = 0.33 95% CI: 0.14–0.77), (30–39) (AOR = 0.35 95% CI: 0.15–0.85), (40–49) (AOR = 0.28 95% CI: 0.10–0.76) respectively, presence of complication (AOR = 2.22 95% CI: 1.36–3.60)**,** prolonged length of hospital stay (AOR = 1.89 95% CI: 1.21–2.95) and having low (AOR = 3.21, 95% CI: 1.66–6.19) and moderate (AOR = 1.99, 95%, CI: 1.14–3.48) social support were factors significantly associated with acute stress disorder.

**Conclusion and recommendation:**

This study showed that the prevalence of acute stress disorder among the adult study participants who experienced traumatic events was high as compared to other literatures. Age, complication, prolonged hospital stay and social support were factors significantly associated with ASD at *p*-value < 0.05. This indicates the need for early identification and interventions or ASD care services from health workers of psychiatric ward.

## Introduction

Acute stress disorder (ASD) is a mental disorder that commonly occur after someone experienced trauma either in the form of direct exposure or witness to the traumatic event or being confronted with events involving actual or threatened death, physical injury, or other threats to the physical integrity of the self or others. Moreover it also includes intense fear, helplessness, or horror responses with the traumatic event within short duration of time [1].

About 20–90% of the general population is exposed to one or more extreme stressful events in their life which causes fear related ASD [2, 3]. Stress diagnoses are drawing attention because of recent increases in traumatic events that occur suddenly such as traffic road accidents, violent,personal assault (like, sexual assault, and physical attack), torture, conflict, terrorism, and natural disaster which may causes Acute stress disorder (ASD). It is characterized by experiencing and avoiding reminders of the traumatic event over time and recurrent, involuntary, and intrusive distressing memories of the traumatic event and dissociative reactions that are higher in traumatic events compared to non-traumatic events [4, 5].

Among traumatic events that are commonly occurred, around 50% of adults will experience a traumatic event in their lifetime and generally prevalence of acute stress disorder among peoples who have experienced trauma ranges from 6–33% [6–9]. Between 21% and 23.6% of adults develop ASD after experiencing a traumatic event [4, 10].

Globally, a systematic review of ASD among traumatized adults showed that the world wide prevalence of ASD in < 1 week post-injury and 1–2 weeks post-injury was: 24.0–24.6% and 11.7–40.6% respectively [11].

According WHO, from 2000–2020 road traffic related deaths would increase with 80% in low and middle income nations, of 32% accounted in sub-Saharan Africa in 2015 [12, 13]. In east Africa including Ethiopia, especially in Sudan and Tanzania in 2014 and 2005 respectively indicates a significant increment of traffic events burden [14–16].

Trauma has numerous negative and psychological impacts on individuals who experienced actual and unexpected serious injury or death [17, 18]. It is also a leading cause of death and disability especially among young adults under 45 years of age and most of them left with serious psychological problems. Individuals who experienced trauma are not exposed only for physical injury it may be causes social, economic, occupational and psychological impact. This results in prolong hospital stay, delay recovery time, increase the cost of care and decrease productivity of the individual following serious injury and unexpected death on the survivors [18–21].

ASD is a hidden problem throughout the world, particularly in countries with a high rate of accident and traumatic event. Ethiopia has a relatively high rate of road traffic accidents when compared to other African countries. According to the Ethiopian National Road Safety Coordination Office, there are 114 road crash fatalities per 10,000 vehicles per year. As a result, early detection is mandatory for better management [22]. However to the researcher knowledge there are insufficient studies available in prevalence of ASD and associated factors in adult age group. Because of inadequate evidence towards ASD,it is difficult to diagnose or treat ASD among adults accurately, compared to other mental disorder (depression or PTSD) [1, 23]. This results in substantial distress, prolonged hospital length of stay and interferes with sleep, social, day to day activities and almost half of the individuals with ASD develop PTSD [24]. This may be because of lack of information and awareness towards ASD especially in low-income developing countries.

Adults are more vulnerable to various traumatic events nowadays, and they should receive early screening, immediate intervention, and ongoing monitoring for ASD to reduce the occurrence of PSTD and suicide. Because evidences showed that patients with ASD are twenty-four times more likely to die by suicide and have a two-fold increased risk of all-cause mortality when compared to the general population.

Understanding the problem could help local decision makers as it helps to design a comprehensive strategy to tackle the problem as early as possible. In addition, for clinicians, it might be helpful to detect and give timely management by identifying the prevalence and factors that are associated with ASD. Furthermore, because there is insufficient research in the study area, this finding provides a baseline for future research on ASD.

Therefore this study aims to assess prevalence of ASD and associated factors among Adults who experienced trauma which is an essential first step toward the plan of early management for clinicians and provides scientific evidence for policy makers, programmers and service planners to make an informed decision that could address the best interests of the patient with ASD which leading to better health and quality of life.

## Methods

### Study design, area and population

An institutional-based cross-sectional quantitative study design was conducted from May– June 2022.The study was carried out in Amhara Comprehensive specialized Hospitals (CSH). According to the 2007 Central Statistical Agency of Ethiopia, Amhara regional state has a total population of 17,221,976 of whom 8,641,580 are male and 8,580,396 females. The Region has eleven administrative zones. There are a total of sixty-seven public hospitals in the region of which eight; Debre Birhan, Debre Markos, Felege Hiwot, Tibebe Geon, University of Gondar, Deretabor, Woldyia and Dessie Hospitals are CSH. Each CSHs serves for 3.5–5 million people [25]. From the eight CSHs the five (Debre Markos, Felege Hiwot, Tibebe Gion, University of Gondar, and Deretabor) are found in northwest of Amhara.

All Adult trauma patients admitted to orthopedic and surgical wards was taken as a source population. All adult trauma patients admitted to orthopedics and surgical ward with trauma of three days and less than one month available during data collection was considered as a study population.

### Variables of the study

**Fig. 1:**
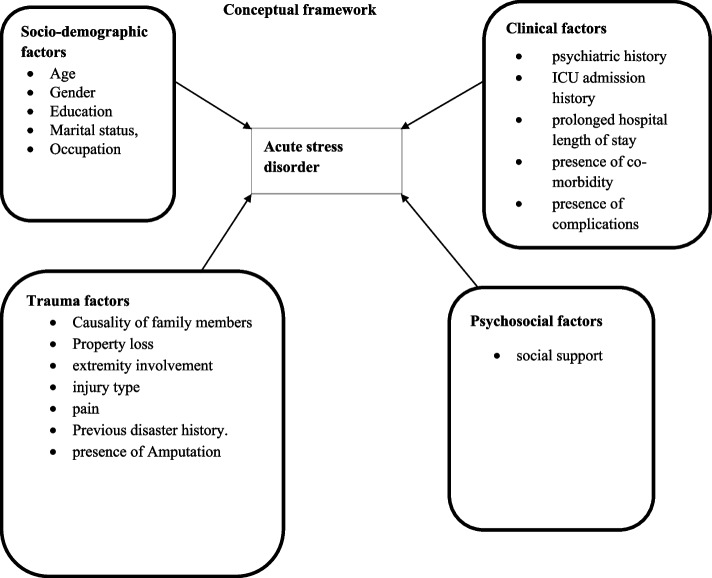
A conceptual framework for factor association with acute stress disorder among Adult trauma patients developed from a review of different literatures [4, 26–31]; CSH-Comprehensive Specialized Hospital

The outcome variable of this study was acute stress disorder. The explanatory variables included in our study were socio-demographic factors (age, educational status, marital status, gender, occupation), clinical factors (psychiatric history, ICU admission history, prolonged hospital length of stay, co-morbidity, complications), trauma related factors (causality of family members, property loss, extremity involvement, injury type, pain, previous disaster history, disaster exposure, presence of amputation) and psychosocial support (social support) (Fig. 1).

### Inclusion and exclusion criteria

#### Inclusion criteria

- All Adult trauma patients admitted to adult surgical and orthopedic wards due to trauma of three days and less than one month was included in the study.

#### Exclusion criteria

- Individuals who have been seriously ill and are unable to respond properly to the questions, as well as those who have been diagnosed with ASD/PTSD prior to trauma with chart review, was excluded.

#### Sample size determination

The sample size was determined by using single population proportion formula considering the following assumptions (Cochran^’^s formula).

$$\text{mi}=\frac{{\left({\text{Z}}_{\text{a}/2}\right)}^{2}\text{ X P}\left(1-\text{P}\right)}{{\text{D}}^{2}}$$

Where

*n* = sample size required for the study

Z = standard normal distribution (z = 1.96) with confidence interval of 95% and

D = 0.05

*P* = the prevalence of acute stress disorder in physically traumatized adults is unknown in our country, hence = 50% (0.5)

D = absolute precision tolerable margin of error (D) = 5% = 0.05

$$n=\frac{{\left(1.96\right)}^{2} X 0.5\left(1-0.5\right)=384}{{\left(0.05\right)}^{2}}$$

Considering 10% non-response rate, nf = 384 + 38 = 422.

#### Sampling techniques and procedure

To select sample of 422 patients from the five referral hospitals, each hospitals was listed down with their respective number of patients, and then the number of patients per month in each hospital was proportionally allocated to sample size; and finally the study subjects of each hospital was selected by using systematic random sampling technique. Based on proportional allocation formula the total sample sizes (422) was allocated to the five public hospitals.

$$\text{nj }= \frac{\text{n XNj}}{\text{N}}$$

**Where;**

Nj = the sample size of the jth hospital

Nj = population size of the jth hospital

n = n_1_ + n_2_ + n_3_ + n_4_ + n_5_ is the total sample size (422)

N = N_1_ + N_2_ + N_3_ + N_4_ + N_5_ is total population size of hospitals (850)

Gondar university comprehensive specialized hospital = 422*250/850 = 124

Tibebe Geon comprehensive specialized hospital = 422*200/850 = 99

Felege Hiwot comprehensive specialized hospital = 422*200/850 = 99

D/Tabor comprehensive specialized hospital = 422*100/850 = 50

**Fig. 2:**
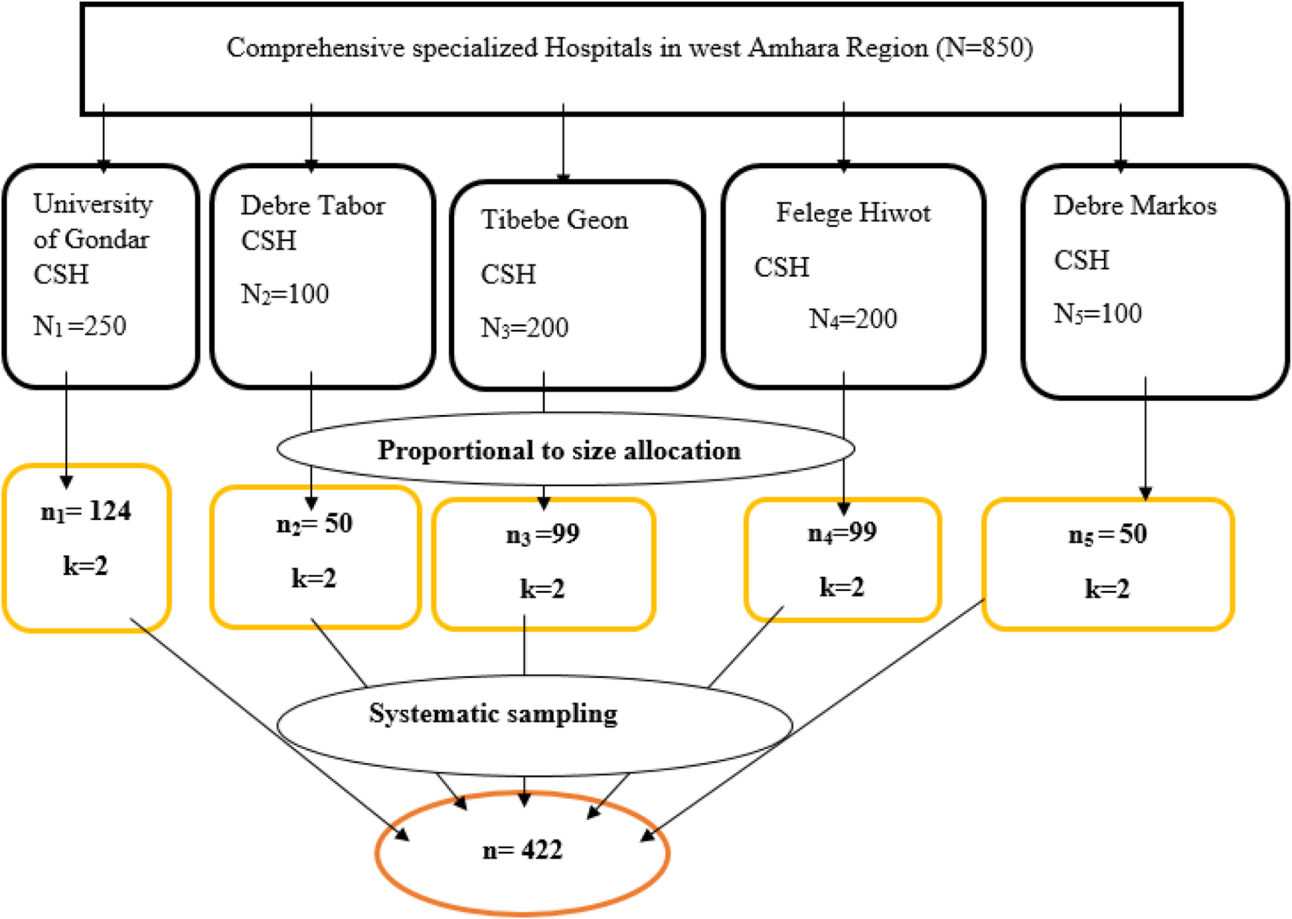
Schematic representation of sampling procedure for prevalence and associated factors of acute stress disorder among adult patients with trauma

D/Markos comprehensive specialized hospital = 422*100/850 = 50 (Fig. 2).

Systematic random sampling technique was applied to select samples from each hospital with the K value determined as follows.

$$k=N/n=850/422=2$$

#### Operational definition

**Trauma (physical injury)** can be explained as a trauma that causes a body wound produced by sudden physical injury from Road traffic accident, Bullet/blast and Blow/assault, and Fall &Crush by a heavy object [7].

**Acute stress disorder** by using DSM-5 those who scored > 9 on the combined re-experiencing(intrusive), dissociative, negative mood, avoidance, and arousal cluster scores, indicates the presence of clinically significant levels of acute stress disorder [32, 33].

**Social support**: According to Multidimensional Scale of Perceived Social Support (MSPSS), participants with mean scale score ranging from 1 to 2.9, 3 to 5, and 5.1 to 7 was considered as having low support; moderate support; and high support, respectively [34].

**Pain**: Operationalized by the 0-to-10 Numerical Rating Scale (NRS) with the values on the pain scale correspond to pain levels as 1–3 = mild pain, 4–6 = moderate pain, and 7–10 = severe pain [35].

**Prolonged hospital length of stay**: a traumatic patient stays in the hospital for ≥ 21 days [36].

**Property loss and causality of family members**; Subjects were asked to self-rate the degree of loss and injury they had experienced in the accident on two 5-point scales. Loss was rated as: 1 = none; 2 = material loss (such as damage to a vehicle or loss of wages); 3 = death of someone unknown; 4 = death of someone known; 5 = death of a relative [4].

### Data collection tool and procedure

A semi-structured pretested questionnaire was used to collect the data. The questionnaire which originally developed in English was translated into the local language (Amharic) by experts and translated back to English to check the consistency. The patients background variables like socio demographic characteristics (such as; age, gender, educational status, marital status and occupation), clinical factors (e.g. Depression) and social support are incorporated in the questionnaire. The questionnaire regarding the trauma related factors, clinical factors and socio demographic characteristics was adapted from different literature’s [4, 29].

Acute stress disorder was measured by using items from DSM-5 diagnostic criteria. The ASD symptom items are listed as “Yes” or “No “questions. The total value with “Yes” on the combined Intrusive, Negative Mood, Dissociative, avoidance, and arousal cluster scores indicates the presence of clinically significant levels of acute stress disorder. In sum, the DSM-5 diagnostic criteria is a valid and reliable self-report instrument for assessing DSM-5 ASD diagnosis for Adults.

Social support was measured by the Multidimensional Scale of Perceived Social Support (MSPSS). Accordingly, participants with mean scale score ranging from 1 to 2.9, 3 to 5, and 5.1 to 7 could be considered as having low support; moderate support; and high support, respectively [34].Its structure and reliability was checked by Cronbach’s alpha (ASD-0.7878, Social support-0.9191).

### Data quality assurance

To maintain the quality of data, data collectors was trained for half day about techniques of interviewing and filling of the check list. In order to measure the validity of check list Pretest was conducted in Dessie comprehensive specialized hospital at April 2022, before the main data collection and which is not included in the final analysis, by taking 5% of trauma patients in a hospital which is out of the study area. The questionnaire was translated from English to Amharic by experts and back to English to assure same meaning is conveyed. Two trained Psychiatry nurses was assigned to collect data through face to face interview for each hospital and 2 Psychiatry nursing professional supervisors was made onsite supervision during data collection period and review all filled checklist during the evening of each data collection day so as to isolate incomplete and incoherent data. In each day evening all filled questionnaire was reviewed and after reviewing incomplete and incoherent questionnaires was isolated.

### Data processing and analysis

Data was checked for completeness and its consistency. Then it was coded and entered into EPI-DATA version 4 and exported into a STATA version 14 for further analysis. Descriptive statistics was computed to describe the frequency, percentages, and distributions of the sample and the result was presented using tables, cross tabulations and charts. The association between dependent and independent variables was assessed by using a binary logistic regression analysis, to estimate the strength of association using Odds Ratios (OR). Multivariable logistic regression was conducted to determine independent predictors of ASD after controlling confounders. All variables associated with ASD with a *p*-value less than 0.2 in the bivariate logistic regression, was further analyzed using a multivariable logistic regression analyses to control potential confounding factors. Variables with a *p*-value less than 0.05 was declared as factors associated with ASD.

## Result

### Socio-demographic status of adult trauma patients in Northwest Amhara comprehensive specialized hospitals, Ethiopia, 2022

A total of (*n* = 422) patients with physical trauma have completed the study with a response rate of 99%. The median age of participants was 28 with age ranging from 18 to 76 years. Out of the study participants, 333 (79.47%) were males, and more than half of participants 210(50.12%) were married. About 170(40.57%) of the participants were farmers and around (32.22% %) of participants were unable to read and write (Table 1).

**Table 1:** Socio-demographic status of adult trauma patients in northwest Amhara Comprehensive Specialized Hospitals, Ethiopia, 2022

Variable	Category	Frequency	Percent
Sex	Male	333	79.47
	Female	86	20.53
Age	18–20	55	13.13
	20–29	171	40.81
	30–39	114	27.21
	40–49	47	11.22
	> = 50	32	7.64
Marital status	Single	181	43.20
	Married	210	50.12
	Divorced/widowed	28	6.68
Educational status	Unable to write & read	135	32.22
	Primary	117	27.92
	Secondary	113	26.97
	Preparatory & college	54	12.89
Occupation	Farmer	170	40.57
	House wife	26	6.21
	Merchant	54	12.89
	Government employed	90	21.48
	Other	79	18.85

N.B: -other includes daily laborer, private, none, tailor, barber, student

### Clinical factors of adult trauma patients in Northwest Amhara comprehensive specialized hospitals, Ethiopia, 2022

Among the study participants about 47(11.22%) had previous psychiatric history in which more than half 25(52.08%) of them accounts for depression, while 148(35.32%) of the respondents had family psychiatric history. Of the participants 86(20.53%) had co-morbidity in which 20(23.26%) and 20(23.26%) were accounts for hypertension and DM respectively. About 33(7.88%) had ICU admission history (Table 2).

**Table 2:** Clinical factors of adult trauma patients in northwest Amhara Comprehensive Specialized Hospitals, Ethiopia, 2022

Variable	Category	Frequency	Percent
Patient psychiatric history presence	Yes	47	11.22
	No	372	88.78
Type psychiatric problem	MDD	24	49.99
	Schizophrenia	7	14.58
	Manic(Bipolar I)	1	2.08
	Dementia	12	25
	Other	3	6.25
Family psychiatric history	Yes	148	35.32
	No	271	64.68
Presence of co-morbidity	Yes	86	20.53
	No	333	79.47
Type of co-morbidity	Hypertension	20	23.26
	DM	20	23.26
	CHF	8	9.30
	TB	10	11.63
	Asthma	26	30.23
	Other	2	2.33
ICU admission history	Yes	33	7.88
	No	386	92.12
Duration in ICU	a week	27	79.41
	2_3 week	5	14.71
	> 3 week	2	5.88

N.B:—others; psychiatric disorder co-morbid with epilepsy, hepatitis (co-morbidity)

*DM* Diabetes mellitus, *CHF* Congestive heart failure, *TB* Tuberculosis, *MDD* Major depressive disorder, *ICU* Intensive care unit

### Trauma related factors of adult trauma patients in Northwest Amhara comprehensive specialized hospitals, Ethiopia, 2022

Among the participants, about 77 (18.38%) had disaster exposure history and more than half 52 (67.53%) was war exposure. For more than half 223 (53.22%) of the respondents, bullet/blast injury was the major cause of trauma. About 329 (78.52%) of them had experienced fracture which accounts 188 (44.87%) on their lower extremity. On the other hand, 50 (62.50%) of the participants had lower extremity amputation and about 140 (38.78%) had moderate level of pain. Among the participants who develop complication 159(37.95%), about 106 (66.25%) were accounts for infection and 201 (47.97%) of them had prolonged hospital stay (Table 3).

**Table 3:** Trauma related factors of adult trauma patients in northwest Amhara Comprehensive Specialized Hospitals, Ethiopia, 2022

Variable	Category	Frequency	Percent
Disaster exposure history	Yes	77	18.38
	No	342	81.62
Disaster type	Floods	23	29.87
	War	52	67.53
	Other	2	2.60
Family/property loss	Yes	33	42.86
	No	44	57.14
Cause of injury	Road traffic accident	92	21.96
	Fall	44	10.50
	Assault	41	9.79
	Machine/crush by object	19	4.53
	Bullet/blast	223	53.22
Affected body part	upper extremity	182	43.44
	lower extremity	188	44.87
	upper and lower extremity	49	11.69
Type of injury	Fracture	329	78.52
	Fracture & Dislocation	35	8.35
	Dislocation & sprain/ligament injury	55	13.13
Complication	Yes	159	37.95
	No	260	62.05
Complication type	infection	105	65.63
	gangrene	34	21.25
	Other	20	12.50
Length of hospital stay	No prolonged hospital stay	218	52.03
	prolonged hospital stay	201	47.97
Amputation	Yes	80	19.09
	No	339	80.91
Amputated part	upper body part	30	37.50
	lower body part	50	62.50
Pain	Yes	361	86.16
	No	58	13.84
Pain intensity	Mild	99	27.42
	Moderate	140	38.78
	severe	122	33.80

N.B: others-ligament injury, nerve injury, numbness, group assault, storm

### Social support of adult trauma patients in northwest Amhara Comprehensive Specialized Hospitals, Ethiopia, 2022

Out of 422 respondents more than half 236(56.32%) of them had moderate social support.

### Prevalence of acute stress disorder among adult trauma patients in northwest Amhara Comprehensive Specialized Hospitals, Ethiopia, 2022

Among 422 study participants the prevalence of ASD among adult trauma patients in Northwest Amhara comprehensive specialized hospitals was found to be 44.15% (95% CI: 39.4%, 49.0%).

### Factors associated with acute stress disorder among adult trauma patients in northwest Amhara Comprehensive Specialized Hospitals, Ethiopia, 2022

To determine the association of independent variables with ASD, bivariate and multivariable binary logistic regression analysis were carried out. In the bivariate analysis factors such as age, affected body part, complication, amputation, pain, prolonged hospital stay and social support were the factors associated with ASD which satisfies a preliminary assumptions (*P* < 0.2 in bivariate logistic regression) to be analyze further in multivariable analysis in order to control potential confounding factors.

Finally, after the variables that passed bivariate analysis were taken in to multivariable logistic regressions for further analysis age, complication, prolonged hospital stay and social support were the factors significantly associated with ASD at *p*-value < 0.05. As a result a respondents age ranging from 21–29 years 77% (AOR = 0.33 95% CI: 0.14–0.77), 30–39 years 75% (AOR = 0.35 95% CI: 0.15–0.85) and 40–49 years were 72% (AOR = 0.28 95% CI: 0.10–0.76) less likely to develop ASD compared to older age group respectively.

Likewise, the odds of presenting ASD among participants who has complication were 2.22 times more likely higher as compared to participants who didn’t have complication (AOR = 2.22 95% CI: 1.36–3.60)**.** Moreover the likelihood of developing ASD was 1.89 times more likely higher among participants with prolonged hospital stay as compared to their counter-part (AOR = 1.89 95% CI: 1.21–2.95).

On the other way, participants who had poor and moderate social support were 3.21 and 1.99 times more likely to develop ASD when compared to participants having good social support(AOR = 3.21, 95% CI: 1.66–6.19) and (AOR = 1.99, 95%, CI: 1.14–3.48), respectively(Table 4).

**Table 4:** Bivariable and multivariable logistic regression of factors associated with acute stress disorder among adult trauma patients in northwest Amhara Comprehensive Specialized Hospitals, Ethiopia, 2022

**Variables**	**Category**	**ASD**		**COR(95% CI)**	**AOR (95% C**I)	***P* value**
		**Yes**	**No**			
Age	18–20	27	28	0.50 (0.20–1.24)	0.38(0.15–1.00)	0.052
	21–29	73	98	0.39(0.18–0.86)	0.33 (0.14–0.77)*	0.011
	30–39	46	68	0.35(0.15–0.80)	0.35(0.15–0.85)*	0.021
	40–49	18	29	0.32(0.13–0.82)	0.28(0.10–0.76)*	0.013
	≥ 50	21	11	1.0	1.0	
Affected body part	Upper extremity	73	109	0.85(0.56–1.28)	1.04 (0.66–1.66)	0.840
	Lower extremity	83	105	1.0	1.0	
	Upper extremity and lower extremity	29	20	1.83(0.97–3.47)	1.04(0.50–2.16)	0.913
Complication of trauma	Yes	94	65	2.68 (1.78–4.03)	2.22 (1.36–3.60)*	0.001
	No	91	169	1.0	1.0	
Presence of amputation	Yes	44	36	1.71 (1.05–2.80)	0.81 (0.44–1.49)	0.506
	No	141	198	1.0	1.0	
Pain	Yes	169	192	2.31(1.25–4.26)	1.88(0.97–3.63)	0.058
	No	16	42	1.0	1.0	
Prolonged hospital stay	prolonged	109	92	2.21(1.49–3.27)	1.89(1.21–2.95)*	0.005
	No prolonged	76	142	1.0	1.0	
Social Support	Poor	54	37	3.51 (1.90–6.48)	3.21(1.66–6.19)*	0.001
	Moderate	104	132	1.89 (1.13–3.18)	1.99 (1.14–3.48)*	0.015
	Good	27	165	1.0	1.0	

ASD acute stress disorder; COR crude odds ratio; AOR adjusted odds ratio; CI confidence interval

*p≤0.05

## Discussion

The prevalence of ASD and associated factors among adult patients with physical trauma in Northwest Amhara comprehensive specialized hospitals were investigated in this study. As a result, it revealed that the prevalence of acute stress disorder among adult patients with physical trauma was found to be 44.15%, which implies it is still the hidden and common community concern that needs early identification and management. The finding of this study is in line with a studies conducted in united Kingdom,south Africa and Ethiopia with 40.6% [37], 40.9% [37] and 45% [38] respectively. This may be related to measurement tools used (DSM-5 criteria).

However, it is significantly lower than a study conducted in Barcelona (Spain) 66.6% [30],New Orleans 62% [39] and Australia 60% [40]. This variation might be due to the differences in instruments, the type of the accident, sample size used and religious adherence to cope from the stress. In Barcelona,Spain it may be due to difference in sample size for only 156 women exposed to a recent sexual assault, in instrument it was DSM-4 and DSM-5 criteria [30]. Similarly in West mead hospital, Australia, the difference may be related to sample size which was for only 51 motor vehicle accident participants using DSM-4 criteria [40]. On the other hand in New Orleans study the difference may be related to sampling method (convenience sampling) among 175 study participants with duration of 2 and 28 days after trauma and study setting which was community-based survey that may increase prevalence cases because of inadequate early treatment and availability of special care with health professions and it was a natural disaster related trauma (flooding) using DSM-4 criteria [39]. Beside to the trauma, in the current study for the increment of prevalence of ASD in Amhara (Ethiopia) may be related to the current crises such as war, conflicts and inadequate human right protection that increases stress.

Conversely, this study is far higher than a studies conducted in united airline(UK), New York, England, two study in China, Brazil and Netherlands that was 25.6%, 24.0%, 21%, 12.59% and 15%, 6.85%, 21.7% respectively [4, 26–28, 41, 42]. Similarly higher than a study done in United Kingdom, John Hopkins burn center, England, Canada with 23.6%, 19%, 14.2% (at week 2) and 3.6% respectively [29, 43–45] and also a study conducted in Belgium and Turkey with 14% and 5(22.7%) respectively [46, 47]. This discrepancy may be related to differences in data collection method, procedures, result scoring method, sample size, type of accident, study setting, measurement tools, socio-cultural, economic difference, poor access to psychiatric counseling, cultural behaviors and inadequate awareness towards acute stress disorder and its treatment between Ethiopia and other countries. In United Kingdom the procedure were involves those participants with road traffic accident who can understand and write English who scored > 35 on the community and in China it was clustered sampling method on disaster exposed participants using DSM-4 criteria. In New York the sample size was 307 using self-administered data collection method and in Brazil the sample size were 146. In addition the sample size in Netherland were 267and in Turk 79 participants with motor vehicle accident using DSM-4.On the other way from the rest of Ethiopian region Amhara regional state were exposed for different forms of traumatic events related to either the geographical area or conflicts in and around the region that results in physical and psychological trauma. Additionally, even though there is good social relationship and religious adherence to cope from the stress, inadequate awareness towards acute stress disorder and its treatment may be contributes for the increment of ASD in the present study.

Regarding age, being a younger age (21–29), (30–39) and (40–49) are less likely to develop ASD compared to older age group (> 50). This was contrary to the previous studies [27, 48, 49].This may be population, nature of the trauma, sample size difference and inadequate researches on older age. Moreover, the difference may be lies on stress management, this might be implies younger people are physically sound to deal with different physical or mental problems. In addition older adults have greater immunological impairment to stress than younger adults, their natural ability to fight back stress gradually decline, increment of chronic illness contracting and developing and delay in wound healing process might be contributes for the development of ASD as their age goes up [50].

In the current study, participants with prolonged hospital length of stay are more prone to develop ASD compared to the counter-part. This is consistent with the study conducted in New York [51]. This may be due to the measurement tool used which was DSM-5. Additionally, these patients represent a significant economic problem on public health systems, their families and the participant also perceive that their discharge delay because of worsening of their case which increases the risk of ASD development.

Likewise participants with complications are significantly associated with ASD than those without complications since it affects them in economy, delays wound healing process and reduce their quality of life which contributes for the outcome occurrence.

Moreover, participants who had low and moderate social support experienced ASD than participants who have high social support. This was affirmed with a study conducted in Denmark and in Ethiopia [38, 52]. This might be related to sample size, instrument such as in Ethiopia it was done among 422 participants using DSM-5 criteria. Moreover even though there is good social relationship and religious adherence to cope from the stress, it might be related to inadequate awareness towards acute stress disorder and its treatment. Likewise those participants with lack of help to compensate for physical incapacity, emotional support and someone to reassure them are might be less likely to cope from trauma related stress which contributes for the development of ASD.

## Conclusion and Recommendation

This study showed that the prevalence of acute stress disorder among the adult study participants who experienced traumatic events was high as compared to other literatures. Age, complication, prolonged hospital stay and social support were the factors significantly associated with ASD at *p*-value < 0.05. The study findings indicated the need for early identification and interventions or ASD care services from health workers of psychiatric ward. Efforts should focus on improving patients' perceptions about waiting in the surgical and orthopedic wards to get adequate care in coordination with psychiatric department. A feasible approach to provide emotional and social support for those who are experiencing prolonged hospital stays.

## Data Availability

All relevant data are available within the manuscript.

## Abbreviations

AOR: Adjusted Odds Ratio
ASD: Acute Stress Disorder
CI: Confidence Interval
COR: Crude Odds Ratio
CSH: Comprehensive Specialized Hospital
DSM: Diagnostic and Statistical Manual of Mental Disorder
ETB: Ethiopian Birr
ICU: Intensive Care Unit
MSPSS: Multidimensional Scale of Perceived Social Support
OR: Odds Ratio
PTSD: Post-Traumatic Stress Disorder
WHO: World Health Organization
